# Basic Pharmacological and Structural Evidence for Class A G-Protein-Coupled Receptor Heteromerization

**DOI:** 10.3389/fphar.2016.00076

**Published:** 2016-03-31

**Authors:** Rafael Franco, Eva Martínez-Pinilla, José L. Lanciego, Gemma Navarro

**Affiliations:** ^1^Departament de Bioquímica i Biomedicina Molecular, Facultat de Biología, Universitat de BarcelonaBarcelona, Spain; ^2^Centro de Investigación Biomédica en Red: Enfermedades Neurodegenerativas (CIBERNED), Instituto de Salud Carlos IIIMadrid, Spain; ^3^Institute of Biomedicine, University of BarcelonaBarcelona, Spain; ^4^Instituto de Neurociencias del Principado de Asturias, Departamento de Morfología y Biología Celular, Facultad de Medicina, Universidad de OviedoAsturias, Spain; ^5^Neurosciences Division, Centre for Applied Medical Research, University of NavarraPamplona, Spain; ^6^Instituto de Investigaciones Sanitarias de NavarraPamplona, Spain

**Keywords:** dimerization, dopamine receptor, GPCR, homodimer, heteromer, ligands, mammalian receptor, signal transduction taste receptor

## Abstract

Cell membrane receptors rarely work on isolation, often they form oligomeric complexes with other receptor molecules and they may directly interact with different proteins of the signal transduction machinery. For a variety of reasons, rhodopsin-like class A G-protein-coupled receptors (GPCRs) seem an exception to the general rule of receptor–receptor direct interaction. In fact, controversy surrounds their potential to form homo- hetero-dimers/oligomers with other class A GPCRs; in a sense, the field is going backward instead of forward. This review focuses on the convergent, complementary and telling evidence showing that homo- and heteromers of class A GPCRs exist in transfected cells and, more importantly, in natural sources. It is time to decide between questioning the occurrence of heteromers or, alternatively, facing the vast scientific and technical challenges that class A receptor-dimer/oligomer existence pose to Pharmacology and to Drug Discovery.

## Introduction

Historical views on any particular topic are both subjective and necessary. In the pre-G-protein-coupled receptor (GPCR) dimer era, pharmacological approaches of a variety of receptors attempted to know GPCR function while providing the tools for developing new drugs targeting GPCRs. Upon cloning, the classical pharmacological approach was challenged by functional studies in heterologous cells transfected with cDNAs coding for receptors. When the possibility of dimer occurrence was first suggested (Fuxe et al., 1983; Fuxe and Agnati, 1985) and, afterward, proved (*vide infra*), the previous working hypothesis and models were unsuitable to provide answers to the new questions. Intriguingly, the field is entering now in an unfruitful controversy instead of facing dimers to change the GPCR molecular physiology and pharmacological paradigms. Around 10 years after the (Prinster et al., 2005) comprehensive review on “*specificity and functional significance”* of GPCR hetero(di)merization and of the first IUPHAR recommendation on recognition and nomenclature of GPCR Hets, there is a need to reinforce the relevance of GPCR heteromerization (class A receptors included) for academic and for pharmaceutical purposes.

Although, GPCR homomerization provides advantages versus single/monomeric receptors (Banères and Parello, 2003; Corriden et al., 2014; Gherbi et al., 2015; Marsango et al., 2015), GPCR heteromerization gives added values for mammalians^1^, namely signaling versatility and diversity. For instance, whereas a dimer of a GPCR coupled to G_i_ would still be coupled to G_i_, a heteromer constituted by two different receptors may couple to different signaling pathways than the individual receptors. Heteromerization in any context, i.e., T cell receptors, taste receptors, or adrenalin, dopamine, adenosine and opioid GPCRs, among others, entail selective advantages. As an example, receptor heteromers (Hets) are needed to taste many different flavors. Should not class A GPCRs heterodimers exist to provide a similar extra-added value (Franco, 2009)? One wonders why evolution could skip this straightforward signal decoding mechanism, but there is enough evidence to show that it is not the case. An exhaustive account of the selective advantages of class A GPCR heteromerization is out of the scope of the present article. From our laboratory we would select the adenosine A_1_–A_2A_ receptor Het, which is a device able to sense the adenosine concentration and respond via G_i_ at low concentrations and via G_s_ at high concentrations (Ciruela et al., 2006; Ferré et al., 2007; Cristóvão-Ferreira et al., 2013). From other laboratories it is very difficult to choose but the coupling of the dopamine D_1_–D_2_ receptor Het to G_q_ (Rashid et al., 2007; So et al., 2007; Hasbi et al., 2009; George et al., 2014) when individual D_1_ or D_2_ receptors are coupled to, respectively, G_s_ or G_i_, is worth mentioning. Also relevant is the finding of opioid receptor Hets that explain the strange pharmacology of opioid receptors and the atypical results obtained by drugs selectively targeting opioid receptor Hets, and that has helped to optimize the opioid receptor nomenclature (Gomes et al., 2000; Portoghese and Lunzer, 2003; Bhushan et al., 2004; Daniels et al., 2005; Waldhoer et al., 2005; van Rijn et al., 2010; Yekkirala et al., 2010; Gupta et al., 2014). Those few examples and the hundreds of already identified Hets contrast with the existing controversy on class A GPCR heteromerization.

Here, we will first compare the little but *convincig* evidence for taste receptor Hets with the similar but *unconvincing* evidence for heteromerization of two class A receptors (dopamine D_1_ and D_2_). Later the review selects a few Hets and a few techniques to build up examples of the varied, complementary and overwhelming evidence of class A GPCR heteromerization.

## Monomeric and Dimeric Taste Receptors versus Evidence for Dopamine D_1_/D_2_ Receptor Hets

The three basic tastes, umami, sweet and bitter, are sensed by two types of specialized GPCRs, taste T1 and T2^2^. T1 and T2 receptors are similar to, respectively, class C and class A GPCRs. IUPHAR indicates that taste T1 receptors are obligate Hets: “*T1R3 acts as an obligate partner in T1R1/T1R3 and T1R2/T1R3 heterodimers, which sense umami or sweet, respectively. T1R1/T1R3 heterodimers respond to L-glutamic acid and may be positively allosterically modulated by 5′-nucleoside monophosphates, such as 5′-GMP [2]^3^. T1R2/T1R3 heterodimers respond to sugars, such as sucrose, and artificial sweeteners, such as saccharin* (Nelson et al., 2001).” In this seminal paper referenced by IUPHAR, Nelson et al. (2001) using an heterologous expression system report “*T1R2 and T1R3 combine to function as a sweet receptor.*” Few years later, Hets for two class A dopamine receptors (D_1_ and D_2_) were identified using a quite similar experimental approach: “*When dopamine D_1_ and D_2_ receptors were coactivated in D_1_-D_2_ receptor hetero-oligomeric complexes, a novel phospholipase C-mediated calcium signal was generated*” (So et al., 2007). For both T1R1/R2/R3 and D_1_–D_2_ Hets, calcium mobilization was used as read-out, transfecting an engineered G protein in the case of taste receptors, and taking profit of endogenous G_q_ expressed in HEK-293T cells in the case of dopamine receptors. In the report of taste receptor heteromerization, transiently transfected cells were used, and in the report of dopamine receptor heteromerization, cells stably expressing D_1_ and D_2_ receptors were employed. Whereas taste T1 and class C GPCRs are considered Hets, class A receptor dimerization in general, or dopamine D_1_–D_2_ receptor Hets in particular are questioned in articles with titles such as: “*GPCR dimers fall apart*” (Lambert, 2010) or “*Evidence against dopamine D_1_/D_2_ receptor heteromers*” (Frederick et al., 2015). Reinforcing the idea of class A GPCR heteromerization is now due.

**FIGURE 1:**
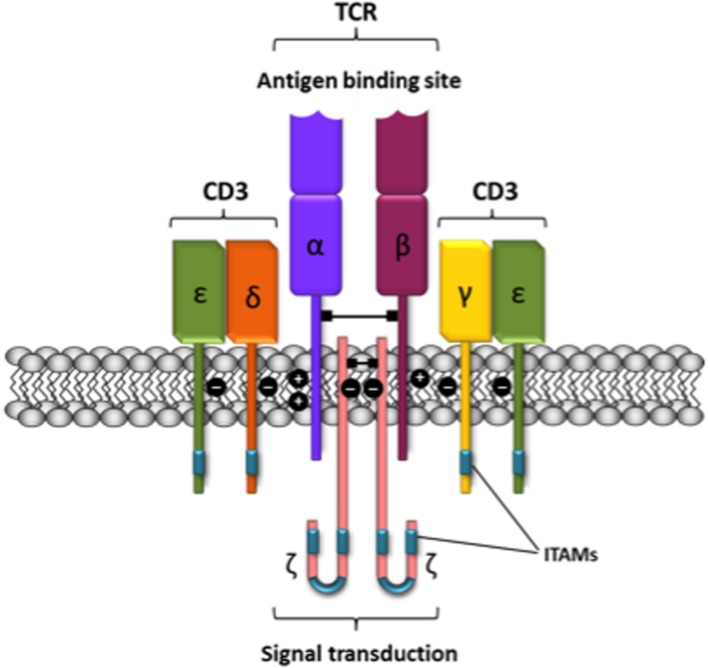
**Schematic representation of the T-cell receptor-CD3 complex.** The heterocomplex is formed by variable TCR-α and TCR-β chains coupled to three dimeric signaling transduction modules CD3δ/ε, CD3γ/ε and CD3ζ/ζ or CD247. CD3, Cluster of differentiation 3; CD247, cluster of differentiation 247 or CD3ζ/ζ; ITAM, immunoreceptor tyrosine-based activation motif; TCR, T-cell receptor.

Class A taste T2 receptors are another example of the criteria used to accept dimerization. Although T2 receptors are often depicted as two molecules interacting together, IUPHAR does not support the dimeric view of these receptors. The main reason for such differential criteria is the big extracellular portion in T1. In fact, the most important difference between class A and C GPCRs is that the last ones have large extracellular agonist binding domains that may dimerize even in the absence of the rest of the receptor molecule (Kunishima et al., 2000; Romano et al., 2001; Kawahara et al., 2012). In contrast, the extracellular N-terminal domain of rhodopsin-like GPCRs is too short to be relevant for GPCR dimer formation. Historically, the discovery of class A GPCR dimers started by taking advantage of techniques used in the Immunology field for detecting interactions between membrane proteins, being the T-cell receptor one of the best studied (**Figure 1**). In this sense, antibody generation and co-immunoprecipitation constituted a revolution in Immunology and served to build up a molecular framework to understand antigen recognition and cell responses. Antibodies raised against GPCRs served to identify GPCRs by immunoblotting, and to label them in cells and tissues. They were also instrumental to identify homo- and heterodimers by immunoblotting and co-immunoprecipitation (Ciruela et al., 1995; Franco et al., 1996; Ginés et al., 2000; Gomes et al., 2000) and, more recently, by proximity ligation assays (Borroto-Escuela et al., 2011, 2012). Other techniques that are not based on antigen-antibody recognition have confirmed these early findings on GPCR homo- and heteromerization.

## The Controversy Surrounding Rhodopsin-Like Class A G-Protein-Coupled Receptor Heteromerization

The hypothesis of dimerization/oligomerization of GPCRs for neurotransmitters was put forward by Fuxe et al. (1983, 1987). However, technical difficulties prevented the discovery of GPCR dimers until the end of the 20th Century. Apart from diverse evidence on receptor homodimerization, and for demonstration that heteromerization was needed for cell surface expression and for GABA_B_ receptor function (White et al., 1998), identification of first class A heterodimers came when Gomes et al. (2000) reported the first Het for two subtypes of opioid receptors μ and δ and when Ginés et al. (2000) identified the first Het of two receptors for two different neurotransmitter/neuromodulators, namely the adenosine A_1_ and dopamine D_1_ receptor Het.

As of 2014 the number of interactions between GPCRs was 537 (Borroto-Escuela et al., 2014). Proved contradictory results constitute approximately 1% of the total Hets, i.e., 5–10, and these exceptions have been reported in a given model but not found in a different model or using another assay type (Fuxe et al., 2014). This very low percentage of inconsistencies substantiates that GPCR and in particular class A GPCR receptor–receptor interactions appear as a robust discovery.

According to the rules of http://www.merriam-webster.com, the belief that GPCRs -or else- are monomers may be referred to as: *monomerism*. The Whorton et al. (2007) report was instrumental for a certain consolidation of this phenomenon. The authors showed that, in an artificial system, monomeric β_2_-adrenergic receptors may activate the coupled G protein. Such article has been frequently used in reviews and in thematic meetings to reinforce the idea that class A GPCRs should be monomers. On the one hand, it is likely that the lipoprotein particles used in the assays could not accommodate two GPCR molecules together to study differential properties of monomers versus homo- or hetero-dimers. On the other hand, an *in vitro* demonstration cannot be used to be certain about receptor operation *in vivo*. Neither the Whorton et al. (2007) data may scientifically substantiate authors’ assertion “*cooperativity of agonist binding is due to G protein association to the receptor monomer and not receptor oligomerization.*” Cooperativity in the binding of ligands to receptor monomers is an oxymoron since this phenomenon requires a dimer or a trimer or a higher-order macromolecule with more than one orthosteric site. In what concerns cooperativity by class A GPCR homomers, two reports using fluorescent ligands to adenosine A_3_ receptors in transfected cells, a challenging technological approach and a solid theoretical formulation and data analysis (May et al., 2011; Corriden et al., 2014), constitute solid grounds to interpret biphasic curves as cooperativity on ligand binding to orthosteric centers in a receptor dimer. Data from radioligand binding assays may provide not only evidence for homomer but for Het formation (Birdsall, 2010). The reluctance to accept class A GPCR dimers may be also challenged taking advantage of both structural information and interpretation of pharmacological data from similarly basic but powerful approaches.

## Are There GPCR Monomers? Structural Data Favoring Class A GPCR Homodimers

The word “monomer,” often used in the GPCR research field is defined by [Encyclopædia Britannica, Inc.] as “*a molecule of any of a class of compounds, mostly organic, that can react with other molecules to form very large molecules, or polymers. The essential feature of a monomer is polyfunctionality, the capacity to form chemical bonds to at least two other monomer molecules. Bifunctional monomers can form only linear, chainlike polymers, but monomers of higher functionality yield cross-linked, network polymeric products.*” On the one hand, this definition of a monomer built on solid chemical knowledge should be considered in the field. On the other hand, even assuming that a given GPCR may act in “isolation” and far from other GPCRs, they are not monomers as they interact, at least, with heterotrimeric G proteins. Therefore, the real issue is whether or not class A GPCRs may interact with other class A GPCRs while they *already* interact with a whole bunch of other proteins. Furthermore, the nomenclature for GPCRs is confusing; probably an appropriate definition of “GPCR molecule” is needed.

The big N-terminal extracellular domain of class C receptors allows formation of crystals constituted by dimers of the extracellular domain; in contrast, the N-terminal domain of class A is very short and unable to form such dimers. This difference in the size of the N-terminal extracellular domain seems to be the only reason that dopamine receptors *may not* form dimers and that class A T2 taste receptors *may not* form dimers whereas class C T1 taste receptors do. Due to the overwhelming evidence for class A GPCR homo/heteromerization, it may well happen that dimerization is guided by transmembrane domains and not by extracellular domains. To our knowledge no study on the relative positions of extracellular versus transmebrane domains has been performed. In the absence of such studies, fixing the dimeric structure of the extracellular domains does not provide any clue on the position of the transmembrane domains of the interacting receptors, i.e., useful information on the quaternary structure of the overall class C GPCR dimer complexes is missing.

Difficulties arising from crystallization of membrane proteins have been sorted out for GPCRs using an approach that, in brief, consists of molecules containing transmembrane helices fused to proteins such as T4 lysozyme or thermostabilized apocytochrome b562, which favor crystal formation. Structures from X-ray diffraction data usually assume a *monomer* as the basic unit. Such interpretation was sanctioned by the finding that arrays of rhodopsin itself in crystals was antiparallel to the plane of the membrane (Edwards et al., 2004; Li et al., 2004), something that is not seemingly physiological but that was not found in other solved rhodopsin structures showing parallel arrangements (*vide infra*).

Subsequent studies, however, have developed crystals in which GPCR dimers exist in the correct -parallel- orientation. The obligate interfaces occurring in 3D GPCR structures (see Cordomí et al., 2015) may not reflect real inter-protomer interfaces of a dimer in its native conformation in the cell surface, but the finding of two neighbor molecules in GPCR crystals compatible with the arrangements of dimers in biological membranes should be considered as relevant. Different transmembrane helices, often in a head-to-head interacting mode (Cordomí et al., 2015) do participate in the parallel receptor dimer arrangement in a variety of class A GPCR crystals, from activated rhodopsin (Salom et al., 2006), to CXCR4 chemokine (Wu et al., 2010) and κ- or μ-opioid receptors (Manglik et al., 2012; Wu et al., 2012). Parallel association of receptors have also been described in crystals of β_2_-adrenoceptors (Cherezov et al., 2007). As Wu et al. (2012) pointed out: “*While the existence of GPCR dimers in vivo and their physiological relevance remain highly debatable, several distinct potential dimer interfaces are starting to emerge from crystallographic and biochemical studies.*”

Noteworthy, μ-opioid receptor bound to a morphinan antagonist crystalizes as a dimer with an interface containing a four-helix bundle motif formed by transmembrane domains 5 and 6 (Manglik et al., 2012). Shortly afterward, the 3D structure of ligand-free turkey β_1_-adrenergic receptors in a lipid membrane-like environment exhibited oligomers constituted by two alternating dimer interfaces, one involving transmembrane helices 4 and 5 and the other engaging transmembrane helices 1 and 2 and the first extracellular loop (Whorton et al., 2013). Last but not least, the seven transmembrane domain of the smoothened SMO receptor crystalizes as a parallel dimer. Despite its relatively small similarity with class A GPCRs, the SMO receptor shows a high degree of spatial conservation of the transmembrane bundle and, also, structural correspondences in the intracellular domains with the solved class A GPCR structures (Wang et al., 2013). These structural features in SMO and class A receptors have led to the suggestion to translate the class A numbering nomenclature (Ballesteros and Weinstein, 1995) to class F GPCRs (Wang et al., 2013).

Lacking yet is any structural data concerning Hets. Precisely, one of the challenges in the GPCR field is Het crystallization, irrespective of whether Hets are constituted by class A or class C receptors. In fact, class C heteromerization has a long way to go; as earlier-mentioned it is not known whether dimerization of the N-extracellular domain conditions the transmembrane intra-dimer interactions. It is possible that the hinge between the N-terminal and the first transmembrane domain loses its conformational flexibility upon dimerization. In other words, it is not known whether transmembrane domains may bring two class C receptors together independently of N-terminal-domain dimerization, i.e., may a given class C Hets have in the membrane more than one quaternary structure? May different dimer structures be established by simultaneously involving either transmembrane regions or the N-terminal domains? Actually, class C Hets raise more structure-related questions than putative class A Hets.

## G-Protein-Coupled Receptors in Heterologous Systems and Knockout Mice

Adrenalin receptors were, and still are, a reference in the GPCR research field. Pioneering studies were centered on adrenergic receptors in natural sources, e.g., isolated heart membranes, but this methodology was almost completely displaced by work in heterologous systems. In fact, COS- or CHO-transfected cells expressing the GPCR have been widespread used to know the whereabouts of these receptors and the scientific advance has been paramount and deserving the 2012 Nobel Prize in Chemistry, awarded to Drs Lefkowitz and Kobilka.

Identification of GPCR dimers occurred in parallel in transfected cells and in samples from natural sources. Biophysical-based methodologies such as fluorescence (FRET) or bioluminescence (BRET) energy transfer have been instrumental to show homo- or hetero-dimers in living transfected cells. A combination of FRET and BRET [sequential resonance energy transfer (SRET); Carriba et al., 2008] has allowed detection of GPCR trimers, and a combination of BRET with molecular complementation, using GPCRs fused to complementary hemiproteins of donor/acceptor BRET pairs, permits detection of tetramers (Cristóvão-Ferreira et al., 2013; Bonaventura et al., 2015). One of the issues raised by *monomerism* is the high-amount of receptors present in transfected cells that may “force” dimer, trimer, etc. formation. By the same token, it could be argued that many of the relevant findings in the last 30 years should be questioned as they have been obtained in heterologous systems overexpressing the receptors. What is more relevant, membranes used in drug screening usually come from GPCR-overexpressing transfected cells that likely express dimer and higher-order structures, therefore, the screening is to, at least, homodimers. Consequently, one may wonder why tools available for receptor dimers, as the two-state dimer model (Franco et al., 2005, 2006; Casadó et al., 2007, 2009a,b), are not used for data analysis; it provides more robust parameters than alternative models such as the two-independent site, which assumes one high affinity and one low-affinity population of *monomeric* receptors. More importantly, should not the previous knowledge be revisited if, as *monomerism* predicts, GPCRs in the heterologous systems are forced to express dimers?

Remarkably, many of the studies describing dimers have attempted and succeeded in proving their occurrence in natural sources. The literature identifying and proving Hets in natural sources is extensive and this review is not intended to exhaustively address it. The occurrence of Hets in natural sources, however, should be taken into account when analyzing data from transgenic animals defective in a given GPCR. GPCRs are somewhat promiscuous in their interaction with other GPCRs^4^ (Borroto-Escuela et al., 2014). Therefore, any particular phenotype in one of these knockout animals is not simply due to the lack of the GPCR but to the lack of the Hets in which the GPCR participates. In this context, pharmacologists may stick to naïvely assign a phenotype to the lack of a *monomer* or take the lead in searching the tools needed to correlate a given phenotype with a particular receptor Het.

## Dimer-Mediated Agonist Affinity Modulation and Cross-Antagonism

Activation of class A GPCRs results in signal transduction, meaning that agonist binding to the GPCR induces conformational changes that are transmitted toward the interior of the cell. Data from radioligand binding assays have confirmed one of the predictions of Het formation, namely the agonist-induced changes of affinity upon binding of a second agonist to the partner receptor in a receptor heterocomplex (Birdsall, 2010). In some cases, such modulation may be of physiological relevance as occurs in the serotonin 5HT_2A_ and metabotropic glutamatergic 2 receptors (González-Maeso et al., 2008). In other cases the phenomenon may reflect dimer formation but the change in affinity is too small to be operating *in vivo*.

Agonist affinity cross-modulation suggests Het formation but it may not reflect a direct receptor–receptor interaction. As G proteins remain attached to GPCRs in the membrane preparations used for radioligand binding, agonist-induced conformational changes in a G protein coupled to one GPCR may indirectly affect a second receptor that is not directly interacting with the first one. By contrast, antagonists and inverse agonists are molecules that bind to GPCRs and inhibit agonist-induced signaling. Very consistently, the selective antagonist of a receptor blocks the signal transduction induced by the agonist binding to the partner heteroreceptor. This counter intuitive fact may be explained by heteromerization. The phenomenon has been described in a variety of GPCR Hets such as orexin/corticotropin-releasing factor receptor (Navarro et al., 2015), dopamine D_1_/histamine H_3_ (Moreno et al., 2011) or angiotensin II AT_1_/dopamine D_2_ (Martínez-Pinilla et al., 2015) receptor Hets. The Ockham’s razor or law of parsimony states “*pluralitas non est ponenda sine necessitate.*” Accordingly, if a convoluted indirect mechanism is not necessary, the simplest explanation is provided by a GPCR Het framework in which the antagonist fixes a non-productive conformational state in one receptor that also impedes the activation of the partner receptor in the Het. Indeed, cross-antagonism should be considered as a tool of heteromer identification. Usually, cross-antagonism is first demonstrated in heterologous cells co-expressing the two receptors and constitutes a so-called *Het print*. Subsequently, dimer occurrence may be investigated on looking for the Het print in samples from mammalian tissues/organs. GPCR-induced activation of the MAP kinase pathway(s) is a successful readout for detecting cross-antagonism, for instance in the central nervous system, both in fresh slices from brain regions and primary cultures of neurons or glia (Balenga et al., 2014; Martínez-Pinilla et al., 2014, 2015).

## Bivalent Probes

The affinity of a given agonist for a given GPCR may change if the receptor is forming Hets (Ferré et al., 2009). Het-selective ligands were then searched for both, detecting Hets *in vivo* and as conceptually novel therapeutic tools. One example is 6′-guanidinonaltrindole, a opioid-receptor-Het selective drug, that in preclinical studies showed analgesia only if administered in the parts on the central nervous system where δ–κ-opioid receptor Hets are expressed. The result was also a proof-of-concept for tissue-selective drug targeting (Waldhoer et al., 2005).

**FIGURE 2:**
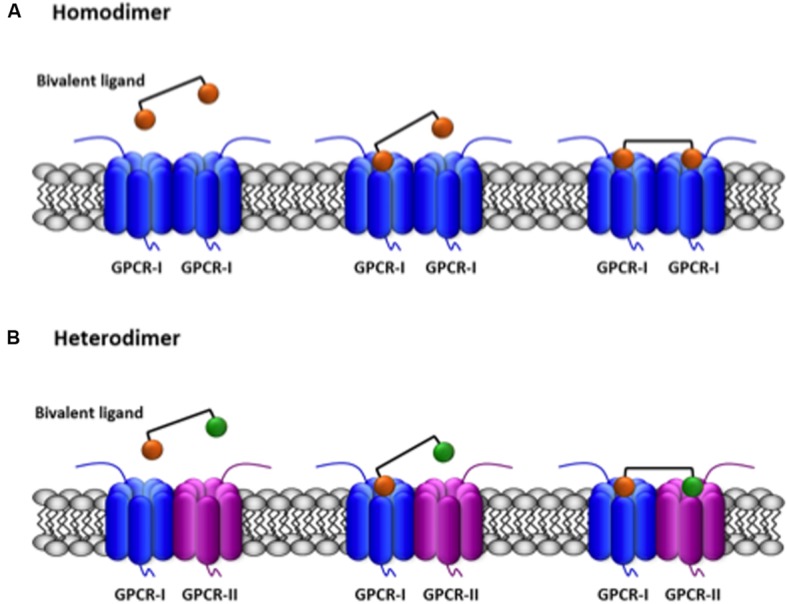
**Scheme of the simultaneous binding of bivalent ligands with linkers of appropriate length to two receptors in a GPCR dimer.** The agonist/antagonist moieties of the bivalent ligand are selective for their respective receptors (orange for GPCR-I, green for GPCR-II) and are linked by an spacer. **(A)** Binding to two equal GPCRs forming an homodimer. **(B)** Binding to two different GPCRs forming an heterodimer. A full account of the possibilities of binding of bivalent ligands to GPCRs is provided by Brogi et al. (2014).

Another strategy of selectively targeting homo- or heterodimers is to synthesize bivalent compounds able to simultaneously bind the two receptors in a GPCR dimer. These molecules consist of two agonist/antagonist moieties separated by spacers of variable length. Therefore, two orthosteric sites may be bridged by a bivalent ligand if the two pharmacophores are appropriately designed. The capacity of simultaneous binding to two different orthosteric sites is an added value that may be used to detect receptor dimers (**Figure 2**). A very detailed review on the various possibilities of bivalent ligands for the characterization of GPCR dimers is provided by Hiller et al. (2013). Certainly, emerging structural information will help revisit data on bivalent compounds to be sure of whether the linkers have the appropriate length to allow simultaneous binding to the two receptors in a given homo- or heterodimer (see Glass et al., 2016).

Synthesis of bivalent ligands for GPCRs was performed in the pioneering report of Halazy et al. (1996), years before GPCR heterodimerization was reported. The authors concluded that their results support: “…*the hypothesis that the important increase in potency of the serotonin dimers can be attributed to the presence of two serotonin pharmacophores in the same molecule.*” Binding of those molecules to one orthosteric site cannot provide any increase in affinity or potency but dimers were not yet seriously considered at that time. It should be also pointed out that as early as in 1982 dimeric peptide enkephalins were synthesized and Shimohigashi et al. (1982) proved their interaction with two δ- but not with two μ-opioid receptors. To explain their results authors took advantage of another phenomenon that, by the way, may occur upon agonist activation, namely receptor clustering (Franco et al., 1996). As dimers for opioid receptors were not yet described, authors assumed that bivalent ligands were simultaneously acting in two close receptors within receptor clusters. Qualitatively similar results were obtained by Carrithers and Lerner (1996) that found bivalent ligands for alpha-melanocyte stimulating hormone receptors more potent than monovalents. Rationale for the study was that “*multivalency can increase the apparent affinity of a ligand for its binding site*” but the authors aimed at designing bivalent ligands “*to bind to two adjacent receptor sites.*” Multivalence of ligands together with dimer occurrence do fit with these pioneering results.

Russo et al. (2007) designed bivalents ligands that were able to bind, in transfected cells, to serotonin 5-HT_4_ constitutive receptor dimers identified by Berthouze et al. (2005). To our knowledge these compounds were not used to address the occurrence of direct 5-HT_4_ receptor–receptor interactions in natural sources. The differential binding to human dopamine D_2_ receptor of bivalent and monovalent ligands also suggested the occurrence of homodimers in transfected cells (Kühhorn et al., 2011a,c). As the theory of ligand binding to receptors would predict, the Hill coefficient of competition curves using a radiolabeled antagonist, [^3^H]spiperone, was 2 for (homo)bivalent and 1 for monovalent molecules (Kuhhorn et al., 2011b).

In Portoghese’s laboratory, the bivalent drug approach was successfully used *in vitro* -in living cells- and in natural sources, but also in behavioral models. Few years after the reporting of the first class A GPCR Hets, bivalent ligands containing δ- and κ-opioid antagonist moieties, provided evidence in the spinal cord of Hets formed by δ-κ-opioid receptors (Bhushan et al., 2004). Remarkably, these Hets explain the atypical pharmacology found for opioid GPCR subtypes in some areas of the central nervous system (Daniels et al., 2005), thus suggesting the occurrence of these receptor Hets in natural sources.

Also consistent with *in vivo* occurrence of μ-opioid/chemokine 5 receptor Hets, selective Het targeting may significantly reduce neuropathic and inflammatory pain (Akgün et al., 2013; Smeester et al., 2014). Although, the therapeutic use of bivalents to combat pain is limited due to the low chances of efficient crossing the blood brain barrier, these results are impotant to confirm Hets occurrence *in vivo*. Performing radioligand binding experiments in samples from tissues, and comparing competition curves of bi- and monovalent drugs could indeed prove or disprove the existence of dimers in mammals. In summary, heterobivalent ligands, i.e., molecules having two different moieties with a linker of appropriate length, are *ad hoc* probes for detecting GPCR Hets in natural sources.

In a detailed and careful study, Soriano et al. (2009) designed, synthesized and tested bivalent ligands containing both adenosine receptor agonist and dopamine receptor antagonist pharmacophores. Molecules with the appropriate spacer length, which was compatible with simultaneous binding to the two receptors, bound (in co-transfected cells) dopamine D_2_ and adenosine A_2A_ receptors with higher affinity than their monovalent counterparts. This finding in cells coexpressing the two receptors was similarly detected in membranes from brain striatum. Those adenosine-dopamine bivalents did not bind with higher affinity to membranes expressing only one of the receptors. Modeling studies indicated that appropriate linker lengths were consistent with simultaneous binding to D_2_ and adenosine A_2A_ receptor dimers. More importantly, the results from competition assays using specific radioligands (for adenosine or for dopamine receptors) can only be explained if there is simultaneous binding to dimers. Basic pharmacology indicates that simultaneous binding of bivalents to two binding sites, should be characterized by higher affinity than the monovalent control compounds. In fact, binding of one moiety to one binding site increases the effective local concentration available to the binding site in a partner receptor.

In summary, despite the field seems not yet mature to fully accept class A GPCR homo/heteromerization, data have been stubborn on providing evidence of the phenomenon. The models used have been very diverse and the techniques have been from co-immunoprecipitation all the way to powerful energy transfer *in vitro* and *ex vivo* assays and *in vivo* transactivation using transgenic mice (Rivero-Müller et al., 2010; Jonas et al., 2013; Grzesik et al., 2014). Class A receptor complexes have also been *visualized* in tissues by means of atomic force microcopy (Fotiadis et al., 2006; Cordomí and Perez, 2009), confocal fluorescence resonance energy transfer (So et al., 2007; Albizu et al., 2010; Perreault et al., 2010; Verma et al., 2010). Moreover, their occurrence, deduced from the avidity of peptides derived from class A GPCRs primary structure to bind together, has been detected by definitive methods^5^ such as mass spectrometry (Ciruela et al., 2004; Navarro et al., 2010), not to mention the positive results from surface plasmon resonance or recently-developed imaging techniques (e.g., fluorescence cross-correlation spectroscopy) able to detect dimer/oligomer-containing single particles.

## Author Contributions

RF: Contacting the Editor and contributing to the writing. JL: Discussing the sections, correcting, and contributing to the writing. GN: Contributing to the writing and searching for some specific references. EM-P: Contributing to the writing and preparing figures.

## Conflict of Interest Statement

The authors declare that the research was conducted in the absence of any commercial or financial relationships that could be construed as a potential conflict of interest.

## Footnotes

1 Actually it would be relevant to check whether heteromerization occurs in invertebrates and/or in lower vertebrates. Finding any animal Kingdom in which class A GPCR Hets do not occur would be extremely relevant to prove heteromerization of class A receptors and to better understand the added value of Hets.

2 www.guidetopharmacology.org

3 Apparently, the linked reference (Li et al., 2002: prostaglandin receptor EP2 mediates PGE2 stimulated hypercalcemia in mice *in vivo*) does not match the information related to taste receptors.

4 www.gpcr-hetnet.com

5 Definitive methods (Uriano and Gravatt, 1977) “are based on a valid and well- described theoretical foundation, which has been experimentally validated so that the reported results have negligible systematic errors and have high levels of precision.”
